# Co-Morbid Disorders in Tourette Syndrome

**DOI:** 10.3233/BEN-120275

**Published:** 2013

**Authors:** Nanette M. M. Mol Debes

**Affiliations:** Paediatric DepartmentHerlev University HospitalHerlev Ringvej 752730 HerlevDenmark

**Keywords:** Tourette syndrome, co-morbidity

## Abstract

Tourette syndrome (TS) is often accompanied by other symptoms and syndromes. The two best-known co-morbidities are Attention Deficit Hyperactivity Disorder (ADHD) and Obsessive Compulsive Disorder (OCD), but also other conditions like rage-attacks, depression, and sleeping disturbances are frequent in persons with TS. Both in clinical cohorts and in population-based cohorts the prevalence of co-morbidities is high. The presence of co-morbid ADHD and/or OCD has an impact on psychosocial, educational, and neuropsychological consequences of TS and it is associated with higher rates of other co-morbid disorders, like rage, anxiety, and conduct disorders. The symptoms of a co-morbid disorder might appear prior to the time that tics reach clinical attention. The TS phenotype probably changes during the course of the disease. The exact aetiology of the co-occurrence of co-morbid disorders and TS is not known, but they probably all are neurotransmitter disorders. European guidelines recommend first-choice pharmacological treatment, but randomised double-blinded trials are needed. Professionals need to be aware of the close relationship between TS and co-morbidities in order to give the patients the right treatment and support.

## 1. Introduction

Tourette syndrome (TS) is a hereditary, chronic, neurobiological disease, characterized by the presence of motor and vocal tics. TS is often accompanied by other symptoms and syndromes, like Attention Deficit Hyperactivity Disorder (ADHD), Obsessive-Compulsive Disorder (OCD), rage attacks, and depression. In this article, the prevalence, impact, course, and treatment of co-morbidities as well as the aetiology will be discussed.

## 2. Prevalence of co-morbidities

### *2.1. ADHD*

ADHD is characterized by difficulties in the ability to focus attention, hyperactivity, and impulsivity. Attention Deficit Disorder (ADD) is a variant of ADHD without hyperactivity. In the general population, 9.2% (5.8%–13.6%) of males and 2.9% (1.9%–4.5%) of females are found to have behaviours consistent with ADHD [1]. In clinical TS populations, the prevalence of ADHD ranges from 21 to 90% (for review see [2]).

### *2.2. OCD*

OCD is characterized by the presence of distressing and intrusive thoughts (obsessions) and repetitive behaviour (compulsions) performed to reduce stress and causes impairment in adaptive functioning and emotional adjustment [3]. Among healthy children and adolescents, the prevalence is 0.5–3.6% [4,5]. Obsessive-compulsive behaviours occur in about 11–80% of persons with TS (for review see [6]).

### *2.3. Rage attacks*

Rage attacks in persons with TS are usually unpredictable, have an explosive quality, and the patients have often a feeling of loss of control. Rage attacks in TS resemble intermittent explosive disorder according to Diagnostic and Statistical Manual of Mental Disorders (DSM-IV) [7]. In clinical TS populations, 25–70% experience episodic behavioural outbursts and anger control problems (for review see [8]).

### *2.4. Sleeping disturbances*

Disturbed sleeping patterns have been reported in 12–62% of persons with TS, varying from nightmares,

ISSN 0953-4180/13/$27.50 © 2013 – IOS Press and the authors. All rights reserved night terrors, somnambulism, trouble falling asleep, restlessness, talking in sleep, early wakening to separation anxiety in the morning (for review see [9,10]).

### 2.5. Depression

Depression and depressive symptoms are found to occur in 13%–76% of persons with TS in clinical cohorts (for review see [11]). Symptoms of seasonal affective disorder seem to occur in persons with TS. In one study, a prevalence of 39.2% was found in a clinical cohort of children with TS [12].

### 2.6. Stuttering

It is often stated that stuttering is quite common in persons with TS, with an estimated incidence of 8–31.3% [13,14]. Abwender et al. [15] have found that developmental stutterers often have tics and comorbidities, like Obsessive Compulsive Behaviour (OCB) and ADHD. Speech of persons with TS is characterized by word repetitions, hesitations, interjections, and prolongations [16,17]. Although it can be difficult to distinguish vocal tics from stuttering in practice, word-medial and wordfinal nonfluencies are indicative of TS and not for developmental stutterers (see for review [18].

### 2.7. Other disorders associated with TS

Several disorders have been suggested to be associated with TS. There are some indications for an association between TS and autism/Asperger syndrome [19–21]. Lawson-Yuen et al. [22] describes a mutation in Neuroligin 4 that may be associated with a wide spectrum of neuropsychiatric conditions, among others TS, autism, and Asperger syndrome. In uncomplicated TS, social cognition seems to be intact, at least on skills involved in empathy and theory of mind [23]. Several studies have described a relationship between epilepsy and TS [24–29]. One of these studies suggests a common dopamine-regulated glutamatergic basis for TS and epilepsy [28]. There are also suggestions that TS is associated with some personality traits, like stubbornness, obstinacy, and inclination to debate [30]. Furthermore, TS is associated with deficits in inhibitory aspects of executive function and real-life-type problem-solving performance is impaired for subjects with TS without a diagnosis of a co-morbid disorder [23,31,32]. Some studies have reported a relationship between TS and schizotypal personality or schizophrenia [33,34]. Other co-morbid conditions described to be associated with TS are separation anxiety, bipolar disorder, pervasive developmental disorder, bizarre behaviours, personality disorders, self-injurious behaviour, and criminal activity (see for review [35]).

## 3. Prevalence studies

The range of the described prevalences of co-morbidities is high. TS populations in the various clinical studies [13,36–41] are inhomogeneous with regard to the age of the included TS subjects. Since the symptoms of both TS and the co-morbidities change over time and with age, this could lead to inconsistencies in the rates found. The design of the various studies also varies between the studies. One could suspect a population-based cohort to have a lower frequency of co-morbidities. Two big population-based studies, however, show that a high prevalence of co-morbidity also is found in community samples.

Apter et al. [43] examined 28,037 individuals aged 16 to 17 years who were screened for induction into the Israeli Defence Force. Twelve of them met the diagnostic criteria for TS (0.04%). Among the persons with TS, 41.7% had OCD compared to 3.4% among those without TS. The rate of ADHD among persons with TS was 8.3% compared to 3.9% in those individuals without TS. The lower prevalence of ADHD in this sample compared to clinical samples of TS subjects is probably caused by the fact that the subjects already were 16–17 years old at the time of examination [43]. Symptoms of hyperactivity usually decline after puberty and therefore many adolescents might not fulfil the DSM-IV criteria to diagnose ADHD [44]. Khalifa et al. [45] examined a total population of 4,479 children aged 7–15 years in Sweden. It was found that 25 children (0.6%) met the DSM-IV criteria for TS. From the population they selected 25 healthy controls. Among those with TS, 68% had ADHD and 16% OCD, compared to 8% ADHD and 0% OCD among the healthy controls [46]. In this sample, the prevalence of OCD is somewhat lower than in clinical TS samples. According to the authors, this can be explained by the fact that the children were young when they were examined and OCD probably starts later in life.

Contrary to these two population-based studies, one recent report described the prevalence of TS, tics, and comorbid ADHD and OCD in a population-based longitudinal study [47]. They suggested that co-occurring OCD is less common in TS cases derived from population-based studies compared with those from clinically ascertained samples. The rate of co-occurring ADHD in the sample was substantially lower than that reported in other population-based studies. This finding might be attributable to the instrument used to diagnose ADHD. Only 8–9% of the TS cases in this study had all three disorders (TS+OCD+ADHD). Nearly 70% did not have either of these two major co-existing conditions. These data suggest that TS individuals in the general population, compared with those seen in specialty clinics, may be more likely to have an isolated tic disorder without ADHD or OCD.

In conclusion, also in population-based studies, the range of prevalence of co-morbid disorders is high and future population-based studies are needed in order to examine this issue more thoroughly.

## 4. Impact of co-morbidity

### 4.1. Other co-morbidities

Several studies have shown that the presence of co-morbid ADHD and/or OCD was associated with higher rates of other co-morbid disorders. Debes et al. [12] described that the frequency of the co-morbidities rage, symptoms of seasonal affective disorder, sleeping disturbances, and depressive symptoms was significantly higher if the co-morbid disorders ADHD and/or OCD were present. Furthermore, more children in the TS+OCD and TS+ADHD+OCD groups stuttered than in the TS-only and TS+ADHD groups, but this difference was not statistically significant. In other studies, co-morbid OCD was shown to be associated with higher rates of anxiety, conduct disorders, and co-morbid ADHD [48,49]. ADHD severity was associated with OCD, learning disorder, and conduct disorder [49]. Comorbid ADHD in children with TS often has a negative impact on concurrent social, academic, and behavioural function, future quality of life, and global psychosocial functioning [50]. Kano et al. [51] found that coprophenomena, impulsiveness/aggression, school refusal, self-injurious behaviours, and clumsiness were more frequent in the groups with co-morbid obsessive-compulsive behaviour and/or hyperkinetic disorder than in the TS-only group.

### 4.2. Severity of tics

The presence of co-morbid ADHD and/or OCD was associated with more severe tics than when these co-morbidties were not present [12,51].

### 4.3. Psychosocial consequences

The presence of co-morbid ADHD was found to be associated with social problems [52] and often interferes in school achievement [53–58]. Debes et al. [59] described higher rates of teasing, social impairments, changing of school, and special education if ADHD and/or OCD were present compared with children with TS without these co-morbidities. Furthermore, the families of the children with ADHD and/or OCD felt lonelier, experienced less understanding in the neighbourhood, and experienced more benefit of contact to other families with TS children than in the group without these co-morbidities [59].

Regarding quality of life, Eddy et al. [60] described that even subjects with TS-only (without co-morbidities) reported worse quality of life (QoL) for general and environmental domains and more depressive symptoms compared to healthy controls. Young people with TS and co-morbid OCD gave lower ratings on the domains of self and relationships than healthy controls. If both co-morbid ADHD and OCD were present, there were found significantly lower scores on all QoL domains in comparison to healthy controls [60].

### 4.4. Neuropsychological consequences

Several studies have examined the impact of co-morbidities on IQ. One study found that the children who belonged to the TS+OCD group scored statistically significant higher on full scale IQ (FSIQ) than the children in the TS+ADHD+OCD group [61]. In that study, there was no statistically significant influence of co-morbidity on verbal IQ (VIQ) or performance IQ (PIQ). Some other studies have confirmed a positive association of obsessive-compulsive symptoms with IQ [62,63]. A number of studies have examined the influence of co-morbid ADHD in subjects with TS and the results are contradictory. Dykens et al. [64] showed that patients with TS+ADHD had lower performance IQ's. Faraone et al. [58] suggested that there might be an ADHD related lowering of FSIQ, but this could not be confirmed by Yeates et al. [54] who found that children with TS and ADHD did not differ from children with TS-only in FSIQ, VIQ or PIQ. One study could not identify a direct relationship between differences in neuropsychological function and differences in ADHD severity or breadth of symptoms [64].

Regarding the examination of the cognitive profile of children with TS, several studies have shown that the presence of co-morbid ADHD was associated with attention deficits, deficits on tasks involving working memory, inhibition, visuomotor integration, and on tasks assessing planning and multitasking ([54, 61,65], see for review [66]). The presence of obsessive symptoms was associated with impaired performance on measures of achievement, executive function, poor recognition memory and inhibitory deficits (see for review [66,67]). Bornstein et al. [68] found a relationship between obsessive-compulsive characteristics and performance on a test sensitive to frontal lobe function. Children with co-morbid ADHD and OCD showed problems in motor tasks and speed tasks [61].

### *4.5. Treatment*

Debes et al. [69] found that in the groups TS+ADHD and TS+ADHD+OCD more children received medical treatment and more different agents were tried compared with the TS-only and TS+OCD groups.

## 5. Aetiology of co-morbidities

The high prevalence of co-morbid disorders in children with TS and the found correlations between tics and co-morbidity confirm the close relationship between TS, OCD, and ADHD. The exact relationship between these three disorders is not clarified yet, but some possibilities are suggested.

First, all three conditions may be considered disorders of disinhibition: TS and OCD express disability to inhibit (in)voluntary repetitive behaviours, and ADHD shows a failure to inhibit socially unacceptable behaviour, verbal responses, and impulsive actions [70]. This failure to inhibit can be explained by frontostriatal dysfunction [71]. There is evidence for involvement of the limbic and orbitofrontal basalganglia-thalamocortical circuits in the pathogenesis of OCD and of the dorsolateral prefrontal and orbitofrontal basalganglia-thalamocortical circuits in ADHD (see for review [70]). Orth et al. [72] found that co-morbid ADHD is associated with more extensive changes in the excitability of motor cortex circuits than uncomplicated TS or the presence of co-morbid OCD. In patients with associated OCD, there was a trend for reduced cortical thickness in the anterior cingulate cortex and hippocampal morphology was altered [73]. In TS patients with associated ADHD there is a volume increase in the dorsolateral putamen [74]. The extent to which various different neuronal circuits are affected might be relevant for the phenotype of Tourette spectrum disorders [72,75].

Furthermore, the three disorders could be related genetically. TS genes may be responsible for an increased susceptibility to ADHD and OCD, perhaps due to neuropathological overlap and/or neurochemical imbalance (see for review [70,76]).

Finally, it has been suggested that the symptoms in pure OCD and pure ADHD can be different from the symptoms in TS+OCD and TS+ADHD, respectively [11,71,76,77]. Concerning ADHD, it has also been suggested that persons with TS might have reduced capacities to maintain attention because of distraction from the tics and the attempts being made to inhibit the tics [4,78]. Furthermore, the psychosocial stress that can arise secondary to co-morbidities might affect the presence of other co-morbidities, as anxiety or mood disorder [48].

Irrespective of the possible relationships, both TS and the co-morbid disorders are presumably neurotransmitter disorders and they often co-occur. Professionals need to be aware of the close relationship between these disorders in order to diagnose the patients correctly and give them the right treatment and support as soon as possible.

## 6. Course of symptoms

Several studies have shown that TS not always starts with tic symptoms. Sometimes the symptoms of a co-morbid disorder appear prior to the time that tics reach clinical attention [45,50,79,80].

If symptoms of ADHD were the presenting symptoms, typically hyperactivity and impulsivity were reported [81]. In general, hyperactive symptoms tend to improve during adolescence, whereas the inattention symptoms often persist in adulthood [82]. OCD symptoms may arise any time during the course of TS. If OCD symptoms were the presenting symptoms, compulsions and not obsessions were reported [81]. Other studies have described that OCD symptoms in patients with TS tend to have an onset around the time that the tics reach their worst-ever, but symptoms may also appear de novo in adulthood [50,82].

Rizzo et al. [75] has examined the long term clinical course of patients with TS in a tertiary clinic. They found that almost half of the children with pure TS at onset changed in TS+OCD phenotype after 10 years follow-up. 48% of children presented ADHD at the onset. After 10 years follow-up this clinical phenotype seemed to disappear. The TS+ADHD phenotype changed to pure TS (62%), TS+OCD (35%), and TS+ADHD+OCD (2%). Mild ADHD symptoms, more inattentive than hyperactive symptoms were still present. Those inattentive symptoms upset the patients less than hyperactive symptoms and anyway less than OCD that became the main problem. 14% of the patients presented the clinical phenotype TS+ADHD+OCD at the beginning and 3 % at follow-up. Rizzo et al. [75] suggested that those subjects who presented with pure TS at onset have a quite good long-term clinical course; by contrast those who presented a co-morbid condition at onset showed a severe prognosis.

## 7. Treatment of co-morbidities

Recently, European guidelines on the treatment of TS have been published [83]. However, in the present literature, there are no big randomized double-blinded trials that examine the pharmacological treatment of TS or co-morbidities. These are needed in the future in order to validate pharmacological treatment.

### *7.1. Co-morbid ADHD*

Behavioural interventions at school and at home are very important in the management of children with TS and concomitant ADHD. If these have insufficient effect, pharmacological treatment should be considered. European guidelines recommend treatment with stimulants, atomoxetine, or clonidine in case of co-existing ADHD. This may be combined with an (antipsychotic) agent for tics [83].

### *7.2. Co-morbid OCD*

European guidelines [83] recommend risperidone as a good first choice in case of coexisting OCD. This may be combined with a serotonin reuptake inhibitor. Given the continuum of tics and obsessive-compulsive symptoms, other agents recommended for the treatment of tics may be tried as well; when partial response occurs, addition of a serotonin reuptake inhibitor or of behavioural treatment may be considered.

### *7.3. Other co-morbidities*

It appears that risperidone is rather effective in reducing the frequency and intensity of rage attacks [84].Clonidine or melatonin might be useful in Tourette syndrome patients with sleeping disturbances [77]. Tricyclic antidepressants and serotonin reuptake inhibitors can be used in patients with co-morbid depression [85].

## 8. Conclusion

TS is often associated with co-morbid disorders, like ADHD and OCD. The presence of these co-morbidities has an impact on psychosocial, educational, neuropsychological consequences of TS and they are associated with a higher rate of other co-morbid disorders, like rage, anxiety, and conduct disorders. The symptoms of a co-morbid disorder might appear prior to the time that tics reach clinical attention. The TS phenotype might change during the course of the disease. Recent European guidelines recommend first-choice pharmacological treatment, but randomised double-blinded trials are needed. The exact aetiology of the co-occurrence of co-morbid disorders and TS is not known, but they probably all are neurotransmitter disorders. Professionals need to be aware of the close relationship between TS and co-morbidities in order to give the patients the right treatment and support.

## Acknowledgements

The author would like to thank Liselotte Skov, M.D., MSc. Pediatric Department, Herlev University Hospital, Denmark for her assistance in preparing this manuscript.
